# Thymoquinone Alleviates Cadmium Induced Stress in Germinated *Lens culinaris* Seeds by Reducing Oxidative Stress and Increasing Antioxidative Activities

**DOI:** 10.3390/life12111779

**Published:** 2022-11-03

**Authors:** Reda Ben Mrid, Abdelhamid Ennoury, Zoulfa Roussi, Imane Naboulsi, Bouchra Benmrid, Anass Kchikich, Redouane El Omari, Mohamed Nhiri, Abdelaziz Yasri

**Affiliations:** 1 AgroBioSciences Research Division, Mohammed VI Polytechnic University, Ben Guerir 43150, Morocco; 2 Laboratory of Biochemistry and Molecular Genetics, FST Tangier, Abdelmalek Essaadi University, Tetouan 93000, Morocco; 3 Higher School of Technology (EST) Sidi Bennour, Chouaib Doukkali University, El Jadida 24000, Morocco; 4 National Institute of Agronomical Research (INRA), Rabat 10100, Morocco

**Keywords:** heavy metals, biostimulant, *Lens culinaris*, germination, carbon and nitrogen enzymes, antioxidant enzymes

## Abstract

This study investigated the effect of thymoquinone on seeds germination and young seedlings of lentils under cadmium (Cd) stress (300 µM). Three different concentrations (10 µM, 1 µM, and 0.1 µM) of thymoquinone were applied. Our results indicated that thymoquinone has a positive effect on several physiological and biochemical parameters on seeds germination and young seedlings of lentils under Cd stress, which led to enhancing their growth. A significant increase in shoot and root length, fresh and dry weight, and chlorophyll content was observed in the treated plants compared to the control plants. However, the thymoquinone treatment significantly reduced malondialdehyde (MDA) and hydrogen peroxide (H_2_O_2_) contents compared to untreated roots and seedlings under Cd-stress. Nevertheless, our results show that the thymoquinone significantly improved the activities of enzymes involved in antioxidant response, including superoxide dismutase (SOD), glutathione peroxidase (GPx), glutathione reductase (GR), thioredoxin reductase (TrxR), and ascorbate peroxidase (APX). We have also studied the activities of isocitrate dehydrogenase (ICDH) and malate dehydrogenase (MDH); ICDH was increased significantly in roots and seedlings in the presence of different doses of thymoquinone. However, the activity MDH was increased only in roots. Our results suggest that the application of thymoquinone could mitigate cadmium induced oxidative stress.

## 1. Introduction

Among heavy metals, cadmium (Cd) is considered a major pollutant in the environment, toxic to plants, animals, microorganisms, and, at certain levels, it may be considered as a threat to human health [1]. The excessive level of Cd also impacts the physiological processes and plant growth of a wide range of crop species [2], suppresses germination, inhibits plant growth and production, and reduces agricultural productivity [3]. A number of studies have reported the adverse impacts of Cd-toxicity in plants, including the destruction of thylakoids, changed chloroplast ultrastructure, and inhibiting enzymes involved in CO_2_ fixation, which affects photosynthesis [4]. At the whole plant level, Cd toxicity increased ROS (Reactive Oxygen Species) production and lipid peroxidation [5,6]. In addition, Cd has become an extremely serious concern for food safety, particularly in the developing countries of the world. 

In order to protect human health, plants, animals, and soil, careful consideration should be given to heavy metals remediation technologies. As a sustainable solution for plants decontamination, we propose the use of plant and microbial secondary metabolites reported to have the ability to mitigate heavy metals phytotoxicity [7]. They were reported to act as potent stimulators of root and shoot developments, as bioprotectant against growth and yield-limiting factors (drought, salinity, temperature, heavy metals phytotoxicity), and also against growth and yield-reducing factors (weeds, suicidal germination, pests, and crop diseases) [7].

Among these biostimulants, the thymoquinone (TQ) (2-Isopropyl-5-methyl-1, 4-benzoquinone) is a secondary metabolite biosynthesized by plants, mainly by the terpene biosynthetic pathway. Thymoquinone is the bioactive constituent of the volatile oil of black cumin (*Nigella sativa* L.) seeds, which are traditionally utilized extensively in the Middle East and in Southeast Asian countries, owing to its various health promoting capacities [8]. TQ exhibits important properties such as antimicrobial, protecting organs against oxidative damage, which is induced by an array of free radical producing agents and the inhibition of membrane lipid peroxidation, and has a significant relevance to the pharmaceutical, cosmetic, agricultural, and food industries [9]. 

Several studies have reported the defensive role of thymoquinone against the harmful effects of heavy metals in animals; however, its role in alleviating Cd-toxicity in plants has been less explored. In a recent study, Ditta et al. [10] reported that exogenous application of black cumin *N. sativa* seed extract improves maize growth under chromium (Cr) stress. In another study, Hussain et al. [5] reported that foliar application of the seed water extract of *Nigella sativa* improved maize growth in Cd-contaminated soil. Seed germination and seedling establishment are critical stages in the life cycle of plant species and the very first phase, which interacts with heavy metals stress [10].

To date, there seems to be no knowledge available in the literature on the effect of TQ to alleviate Cd-induced stress in germinated lentil seeds. For this reason, this work investigated the impact of TQ in reducing the effects of Cd on the seed germination and seedling growth of lentil. Therefore, our objectives were to (i) evaluate Cd-stressed lentil seed germination and lentil seedling parameters using TQ (ii) evaluate the protective role of thymoquinone under Cd toxicity conditions by improvement of antioxidant defense enzymes such as superoxide dismutase, glutathione reductase, glutathione peroxidase, thioredoxin reductase, and ascorbate peroxidase and enzymes implicated in nitrogen and carbon metabolism enzymes, such as isocitrate dehydrogenase and malate dehydrogenase.

## 2. Material and Methods

### 2.1. Germination Conditions and Treatments

Firstly, the healthy and robust lentil seeds (*Lens culinaris*) were surface disinfected with 2% (*v*/*v*) sodium hypochlorite for 10 min, washed thoroughly, and soaked in distilled water at 4 °C for 30 min. We had 4 replicates for a total of 5 treatments and considered 25 seeds in each treatment. Consequently, we calculated 5 ∙ 4 ∙ 25 = 500 seeds. Cadmium sulfate (CdSO_4_) as salt of cadmium was used to prepare the desired cadmium concentration. One level of Cd (300 µM) was used in the experiment along with control (without Cd). Twenty petri dishes (four replicates for five treatments) were washed with deionized water and lined with filter paper (Whatman) for the germination study. Each petri dish (10 cm) received 25 lentils seeds, and then they were placed in the dark for one day in an air-conditioned room at 25 °C. Later, Petri dishes were transferred to light at a flux density of 150 μmol/(m^2^ s), and were treated daily with 2 mL with one the following conditions: (1) H_2_O (control), (2) Cd (300 µM), (3) Thymoquinone (10 µM) + Cd (300 µM), (4) Thymoquinone (1 µM) + Cd (300 µM), and (5) Thymoquinone (0.1 µM) + Cd (300 µM). The petri dishes were kept for eight days in an air-conditioned room at 25 °C with a day/night cycle of 12 h/12 h. 

Plant seedlings were harvested on the ninth day after treatment and germinated seeds were counted daily based on 2 mm radicle emergence for up to three days.

### 2.2. Measurements of Germination Parameters

The daily count of germinated seeds (GP) was performed every 24 h for 3 days in each experimental unit, and seeds with a seminal root length of more than 2 millimeters were counted as germinated seeds (ISTA, 2009), which was determined using the following formula: GP = (number of germinated seeds/total number of seeds) × 100.

After a period of time necessary for planting seeds (9 days), 10 seedlings were selected in each Petri dish. The length of the seminal root and shoot was measured with the ruler and then, the roots and shoots were separated and the fresh weight was immediately measured and placed in an oven at 50 °C for 72 h. After drying the shoots and roots, they were weighed per treatment individually.

In this experiment, the germination index (GI) was calculated as described by the Association of Official Seed Analysts (AOSA 1983) using the following equation, GI = ∑(Gt/Tt), where Gt is the number of seeds germinated on day t and Tt is the number of days, and the seedling vigor index (SVI), described by the following formula: SVI = (seedling length (cm) × germination percentage).

### 2.3. MDA and H_2_O_2_ Contents

The malondialdehyde (MDA) content in the roots and aerial parts of lentil seedlings was determined using the protocol mentioned by Ennoury et al. [11]. The roots and aerial parts of lentil seedlings (0.4 g) were ground in 5.0% (*w*/*v*) trichloroacetic acid (TCA) and subsequently centrifuged at 12,000× *g* for 10 min at 4 °C. The supernatant was collected in a screw-capped tube, followed by the addition of 300 µL of trichloroacetic acid (20%) and TBA (0.67%). The mixture was heated at 95 °C for 1 h. After cooling, 1 mL *n*-butanol was added to the mixture followed by centrifugation at 12,000× *g* for 10 min. Organic supernatant was collected to measure the absorbance at 532 nm. The H_2_O_2_ content was determined in the roots and aerial parts of the lentil seedlings following the method of Velikova et al. [12], with some modifications. Roots and aerial parts of lentil seedlings (0.1 g) were ground with 1.0 mL of 0.10% TCA and centrifuged at 11,500× *g* for 12 min at 4 °C temperature. The 0.5 mL supernatant was taken in a test tube, 10 mM potassium phosphate buffer (pH 7.0) (0.5 mL) and 1.0 M potassium iodate (1.0 mL) were added, and the mixture was incubated in the dark for 60 min. The absorbance was quantified at 390 nm.

### 2.4. Enzymatic Measurements

#### 2.4.1. Extraction and Assay of SOD, GR, GPx, TrxR and APX

A total of 0.2 g of fresh roots and aerial parts of the lentil seedlings were extracted using a mixture containing the following: 100 mM HEPES-KOH, 20 µM FAD, 10 mM MgCl_2_, 1 mM PMSF, and 14 mM β-Mercaptoethanol. The homogenate was centrifugated (20,000× *g*) for 20 min at 4 °C. The supernatant was used to determine the enzyme activities. The activity of SOD was assayed according to the method described by Ben Mrid et al. [13]. The GR activity was determined by the oxidation of NADPH at 340 nm, as described by El Omari et al. [14]. The GPx activity was carried out according to the method described by Bouchmaa et al. [15], with some modifications. The reaction mixture contained 0.05 M (instead of 0.1 M) potassium phosphate, pH 7.0, 1 mM EDTA, 1 mM sodium azide, 1 mM GSH, GR (10 μg/mL), 0.25 mM NADPH, 0.25 mM of H_2_O_2_, and enzyme extract. The TrxR was carried out according to the method described by Ben Mrid et al. [16]. The APX activity is measured by the following method adapted from Nakano and Asada [17]. 

#### 2.4.2. Extraction and Assay of NADH-MDH and NADP^+^-ICDH

The activities of NADH-MDH and NADP^+^-ICDH were extracted and assayed according to the method described by Ben Mrid et al. [18] and Ben Mrid et al. [19], respectively.

### 2.5. Estimation of Chlorophyll

The extraction of aerial parts of lentil seedlings chlorophyll was performed with 80% acetone. The chlorophyll a, chlorophyll b, and total chlorophyll quantities were calculated according to the method of Arnon [20].

### 2.6. Estimation of Protein

The roots and aerial parts of lentil seedlings after measurements of germination parameters were ground in a mortar with pestle in an extraction buffer containing: 100 mM HEPES-KOH, 20 µM FAD, 10 mM MgCl_2_, 1 mM PMSF, and 14 mM β-Mercaptoethanol. The resulting homogenates were centrifuged twice at 15,000× *g* for 30 min at 4 °C and the supernatant was removed to a new tube. The total soluble protein content was determined following the method of Bradford [21], using the Bradford reagent (Sigma Aldrich, St. Louis, MO, USA) and bovine serum albumin (BSA) as a protein standard.

### 2.7. Statistical Analysis

SPSS 25 package for Windows, version 10.0.1, was used for all statistical analyses. ANOVA one factor, followed by the Student Newman–Keuls post hoc test, were used to compare differences in the means (*p* < 0.05). Different letters indicate significant differences.

## 3. Results 

### 3.1. Effect of Thymoquinone on Seed Germination Parameters in Lentil Seeds Germinated under Cd Stress

Lentil seeds exposed to Cd stress have shown a significant decrease in germination percentage by 30.6%, 17.6%, and 16% in the 1st, 2nd, and 3rd day of germination, respectively, compared to the control. Cd exposure has also decreased the germination index (GI) and seedling vigor index (SVI) significantly by 23% and 65%, respectively, compared to the control (Table 1). Thymoquinone treatment on lentil seeds under Cd stress has neutralized the Cd effect on germination percentage after three days of germination. Treatment with 10 µM, 1 µM, and 0.1 µM of thymoquinone increased the GP by 22%, 20%, and 19%, respectively, compared to seeds treated with Cd only (Table 1). Furthermore, treatment with 10 µM, 1 µM, and 0.1 µM of thymoquinone have increased both GI (by 25.43%, 34.1%, and 28%, respectively; Table 1) and SVI (by 62%, 65.5%, and 66%, respectively; Table 1) of lentil seeds compared to Cd-treated only.

### 3.2. Effect of Thymoquinone on Growth Parameters and Chlorophyll Content in Lentil Seedlings Germinated under Cd Stress

In our results, shoots and roots length, the fresh and dry weight decreased significantly compared to the control (Table 1). Indeed, the shoots and roots lengths were decreased by 36.99% and 77.83% compared to the control, respectively. The shoots and roots fresh weights were reduced by 46.66% and 78.48%, respectively, compared to the control. Moreover, the shoots and the roots dry weights were reduced by 49.12% and 68.82%, respectively, compared to the control (Table 1). Nevertheless, treatment with 10 µM, 1 µM, and 0.1 µM of thymoquinone have an increased shoot length (by 36.59%, 35.31%, and 37.87%, respectively), fresh weight (by 49.87%, 38.59%, and 41.82%, respectively), and dry weight (by 79.90%, 59.80%, and 54.41%, respectively) of lentil seedlings compared to shoots with treated Cd (Table 1). The same behavior has been observed for roots length, fresh weight, and dry weight (Table 1). 

**Figure 1:**
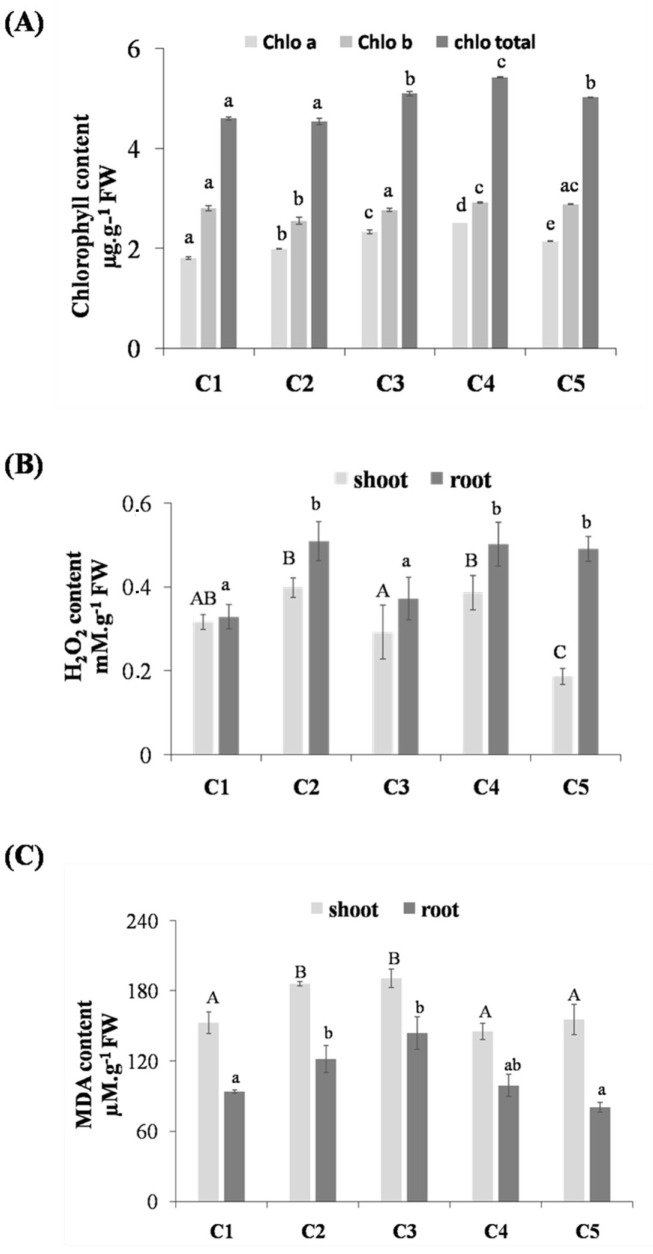
Chlorophyll contents (**A**) in shoot and H_2_O_2_ (**B**) and MDA (**C**) contents in shoots and roots of germinated lentil treated by different concentrations of thymoquinone. C1: treated with water (Control); C2: treated with cadmium; C3: treated with thymoquinone 10 µM + Cd; C4: treated with thymoquinone 1 µM + Cd; C5: treated with thymoquinone 0.1 µM + Cd. Each value represents the mean of four independent observations ±SD. Means with the same letter are not significantly different at the 5% probability.

Our data showed that the content of chlorophyll (a) was increased by 10% in plants treated by Cd compared to the control. While the chlorophyll (b) content showed a weak decrease under the Cd-stress compared to the control, the chlorophyll total seems unaffected in the presence of Cd (Figure 1A). The treatment with thymoquinone has significantly stimulated the pigment content under Cd stress. For example, the treatment with 10^−6^M of thymoquinone had significantly increased the chlorophyll (a), chlorophyll (b), and chlorophyll total content by 26.26%, 14.17%, and 19.42%, respectively, compared to the control plants.

### 3.3. Effect of Thymoquinone on H_2_O_2_ and MDA Contents in Lentil Roots and Shoots under Cd Stress

In our study, the H_2_O_2_ and MDA contents were increased in plants treated with Cd compared to the control (Figure 1B,C). The H_2_O_2_ content significantly increased in shoots and roots by 25.8% and 56.25%, respectively, compared to the control. The same behavior was observed in the case of MDA content, which shows a significant increase of 21.95% and 29.75% in the shoots and roots of plants treated by Cd compared to the control. The treatment with thymoquinone decreased the MDA and H_2_O_2_ contents obviously at 1 µM and 10 µM, respectively. Thus, the H_2_O_2_ content was decreased by 25.64%, and 26% in shoots and roots, respectively, in plants treated with 10 µM of thymoquinone. Meanwhile, the MDA content decreased by 22.04% and 18.63% in shoots and roots, respectively, in plants treated with 1 µM of thymoquinone.

### 3.4. Effect of Thymoquinone on Activities of Antioxidant Enzymes in Lentil Roots and Shoots under Cd Stress

**Figure 2:**
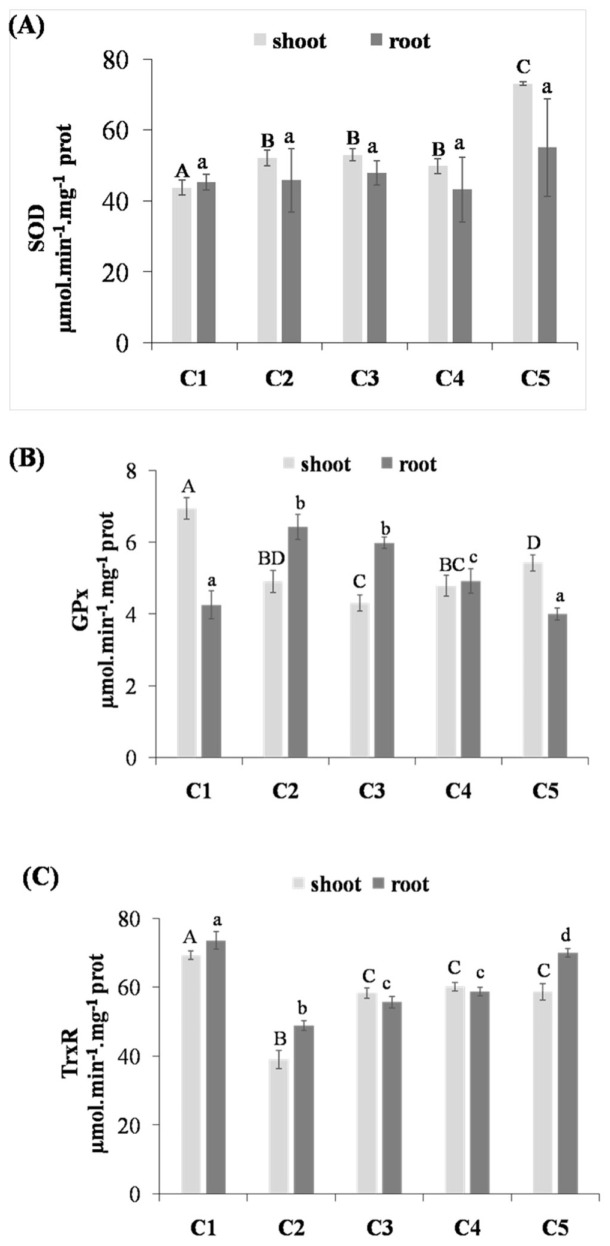
Activities of superoxide dismutase (SOD) (**A**), glutathione-peroxidase (GPx) (**B**), and thioredoxin-reductase (TrxR) (**C**) in the shoots and roots of germinated lentils treated by different concentrations of thymoquinone. C1: treated with water (Control); C2: treated with Cd; C3: treated with thymoquinone 10 µM + Cd; C4: treated with thymoquinone 1 µM + Cd; C5: treated with thymoquinone 0.1 µM + Cd. Each value represents the mean of four independent observations ±SD. Means with the same letter are not significantly different at the 5% probability.

In this study, the SOD activity increased moderately in lentil shoots and roots exposed to Cd compared to the control. In thymoquinone-treated plants, and especially at the concentration of 0.1 µM, the activity of SOD increased by 66.69% and 21.41% in shoots and roots, respectively, compared to the control, and by 40.03% and 20.17% in shoots and roots, respectively, compared to the Cd-treated seedlings (Figure 2A). The GPx and TrxR activities in treated plants with 10 µM of thymoquinone has not been significantly changed compared to the Cd stressed plants without treatment. However, at a concentration of 0.1 µM, the TrxR regained their normal activities, similar to that observed in the control plants (Figure 2B,C).

**Figure 3:**
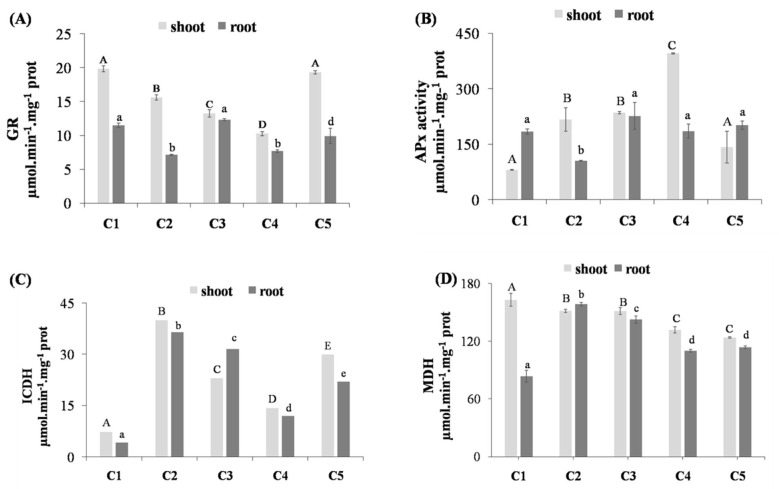
Activities of glutathione reductase (GR) (**A**), ascorbate peroxidase (APx) (**B**), isocitrate dehydrogenase (ICDH) (**C**) and malate dehydrogenase (MDH) (**D**) in the shoots and roots of germinated lentil treated by different concentrations of thymoquinone. C1: treated with water (Control); C2: treated with Cd; C3: treated with thymoquinone 10 µM + Cd; C4: treated with thymoquinone 1 µM + Cd; C5: treated with thymoquinone 0.1 µM + Cd. Each value represents the mean of four independent observations ±SD. Means with the same letter are not significantly different at the 5% probability.

In this study, the GR (Figure 3A) activity has decreased in both lentil shoots and roots compared to non-stressed plants. However, the use of 0.1 µM of thymoquinone has significantly increased these enzyme activities in both organs to a close level to that observed in non-stressed plants. Our results also showed an upregulation in APx activity in lentil shoots under Cd-stress, while a decrease in roots was observed. The APx activity was increased by 168.14% in shoots treated with Cd compared to the control plants, while a decrease by 42.69% was found in roots under the same condition compared to the control (Figure 3B). The treatment by thymoquinone has significantly increased the activity of APx compared to the plants treated by Cd. Thus, the treatment by 1 µM of thymoquinone presents the highest value of APx activity, which was stimulated by 82.02% in shoots and 75.44% in roots compared to the control plants.

### 3.5. Effect of Thymoquinone on Activity of ICDH and MDH in the Lentil Roots and Shoots under Cd Stress

In our study, the NADP^+^-ICDH activity was also influenced by the Cd stress in the roots and shoots of lentil (Figure 3C). Furthermore, the enzyme activity increased by more than 445% in the shoots and by more than 767% in the roots compared to the control plants. However, the thymoquinone treatment led to a significant decrease in the NADP^+^-ICDH activity in both organs. When exposed to stress Cd, the MDH activity in lentil roots increased significantly to reach 158.61 µmol∙min^−1^∙mg^−1^ of protein compared to shoots control with 83.37 µmol∙min^−1^∙mg^−1^ of protein. The thymoquinone-treated plants showed a significant decrease in MDH activity in the roots, especially those that were treated with 1 and 0.1 µM, for which the activity was 110 µmol∙min^−1^∙mg^−1^ of protein and 113.64 µmol∙min^−1^∙mg^−1^, respectively (Figure 3D).

## 4. Discussion

### 4.1. Effect of Thymoquinone on Seed Germination Growth Parameters and Chlorophyll Content in Lentil Seeds Germinated under Cd Stress

Our results showed that lentil seeds exposed to Cd stress have shown a significant decrease in germination, shoots and roots length, and in the fresh and dry weight compared to the control. These results are in agreement with those obtained by Tauqeer et al. [4], who found that a high Cd concentration (2.0 mM) resulted in a decrease in *Alternanthera bettzickiana* plant growth and biomass. Vijayaragavan et al. [22] indicated that the inhibitory effect of Cd in seeds of cowpea (*Vigna unguiculata* L.) is due to a deficiency of water absorption, which causes a reduction in the supply of water necessary for seed embryo development. The application of TQ treatment in lentil seeds under Cd stress has been shown to neutralize the Cd effect by increasing the germination percentage after three days of germination. These findings are in agreement with previous studies, suggesting the beneficial effect of plants biostimulants on seed germination and seedling parameters such as height, fresh, and dry weight by minimizing the availability of Cd and its accumulation and toxicity [23].

In the presence of Cd, our result showed that the chlorophyll total was not affected despite the increase of chlorophyll (a) content and the decrease of chlorophyll (b) content. The increases in the ratio of Chl a:b have been linked with the change in pigment composition of the photosynthetic apparatus. which possesses a lower level of light harvesting chlorophyll proteins (LHCPs) [24]. The reduction in LHCPs content is an adaptive defense mechanism of chloroplasts, leaves, and plants, which allows them to endure the adverse conditions [25]. The treatment with TQ stimulated the chlorophyll content in the treated plants. This increase in chlorophyll content could be the result of a reduction in chlorophyll degradation [14]. The increase of chlorophyll content in plants treated with TQ can be considered as an index of Cd alleviation in plants.

### 4.2. Effect of Thymoquinone on H_2_O_2_ and MDA Contents in Lentil Roots and Shoots under Cd Stress

High concentrations of MDA and H_2_O_2_ contents in Cd-stressed lentil seedlings is a clear indication that the Cd enhances free radical production, as previously mentioned in several studies. Muthukumar et al. [26], who studied the effect of Cd on the antioxidant status of *Saccharomyces cerevisiae* as a model for the molecular mechanisms of oxidative stress, found that the concentration of MDA significantly increased in Cd-exposed cells compared to unexposed ones. Sakouhi et al. [27] have found an increased in H_2_O_2_ content in chickpea seedlings treated with Cd. Cd toxicity inhibits the electron transfer, which causes inhibition in the photo-activation of photosystem II [28]. Therefore, Cd may indirectly contribute to the production of ROS through perturbation in the chloroplasts of leaves [29]. The decrease in the MDA and H_2_O_2_ contents observed in our study in the presence of TQ can be considered as a good index for the antioxidant effect of TQ against Cd stress. In fact, the chemical structure of TQ is the key to its antioxidant activity. Its chemical structures contain a phenol ring, double bonds, and hydrogen atom (H+), allowing for free delocalization of electrons and H^+^ donation, in which the scavenging and neutralization of ROS attenuates the oxidative damage [30]. Previous studies have proposed various implied mechanisms for the antioxidant potential of TQ, which includes forthright interaction of TQ with GSH or NADP to form thymohydroquinone or glutathionyldihydro-TQ complexes that are able to quench ROS and boost the expression of antioxidant genes [31,32]. 

### 4.3. Effect of Thymoquinone on Antioxidant Enzymes in Lentil Shoots and Roots under Cd Stress

Superoxide dismutase (SOD) is known to be the first defense barrier against ROS. This enzyme dismutates the superoxide anion radical (O_2_^−^) to hydrogen peroxide (H_2_O_2_). The subsequent H_2_O_2_ is then neutralized by its conversion to H_2_O in a reaction catalyzed by either CAT or GPx. The results of the present study have shown an increase in SOD activity under Cd stress in roots, and the same result has been reported in several studies [33]. In this study, it was shown that GPx activity decreased in the shoots but increased significantly in the roots under Cd-stress condition compared to the control. Ammar et al. [34] reported that the GPx activity increased significantly in Cd stressed tomato plants, however, it decreased in tomato leaves treated with Cd concentrations ranging from 1 to 25 µM and increased at 50 µM. Thymoquinone is well reported for its activity as an antioxidant at low concentrations. However, at high concentrations, TQ has been reported to act as a prooxidant [35]. In this case, at a low concentration (10^−7^), the TQ acted as an antioxidant and reduced the ROS generation, which led to a balance of the enzyme activities. However, further analysis should be conducted to validate this hypothesis. Thymoquinone treatment has been shown to reduce oxidative stress markers (superoxide and hydrogen peroxide) and attenuate oxidative stress by promoting antioxidant enzymes (SOD, GPx, TrxR, GR, and APX), and the GSH level, downregulating pro-oxidant genes and upregulating antioxidant genes [36].

The Asada–Halliwell pathway is the major pathway of antioxidant defense, which mainly detoxifies the H_2_O_2_ in a plant cell [37]. Apart from AsA and GSH, its enzymes—APX, MDHAR, DHAR, and GR [38]—have significant roles. AsA and GSH are strong antioxidants and have high redox potentials, which is important in conferring the stress tolerance in plants [38]. Therefore, the higher APX and GR activities observed in the presence of TQ might play a function in scavenging excess ROS by production of GSH and AsA. It has been reported that the ascorbate regulates phytohormone biosynthesis, consequently modulating plant development [39,40]. Furthermore, a number of dioxygenases that are directly related to hormonal biosynthesis require AsA as a cofactor [41]. On the other hand, 1-aminocyclopropane-1-carboxylate (ACC) synthase (ACS) and ACC oxidase (ACO) genes encoding ethylene biosynthetic enzymes is induced by GSH [37], which explains the increased growth in the presence of TQ.

The redox homeostasis and the antioxidant capacity of the cells are also maintained by the pool of the thioredoxin [42]. Moreover, Pandey et al. [43] have found that the enhanced expression of TrxR helps in mitigating oxidative stress in arsenic-treated *Anabaena*. Thus, under Cd stress, the low activity of TrxR enzymes is also correlated with the incapability of the plants to reduce oxidative stress. The increase in the enzyme activity TrxR after the application of TQ at low concentrations (10^−7^ M) may indicate a protective role of this compound against oxidative stress. 

### 4.4. Effect of Thymoquinone on Activity of ICDH and MDH in Lentil Roots and Shoots under Cd Stress

Both thioredoxin- and glutathione-dependent systems necessitate NADPH, as a cofactor, to be active against ROS generated by the oxidative stress [44]. Accordingly, low levels of NADPH may have a negative impact on the cell response to ROS. Moreover, the depletion of NADPH under oxidative stress conditions has already been demonstrated and is suggested to be due to the higher demand for NADPH by NADPH-dependent antioxidant enzymes [45]. One of the main enzymes involved in the provision of NADPH is the NADP^+^-ICDH [44]. Indeed, it was stated that the over-expression of this enzyme correlated with high resistance to oxidative stress, and that low levels of NADP^+^-ICDH are responsible for a low resistance to oxidative stress [45]. The high increase in the NADP^+^-ICDH activity under Cd stress may be related to the high demand for NADPH to cope with the oxidative stress. However, as the TQ has an antioxidant effect, the demand for NADPH decreased, which may explain the decrease in the NADP^+^-ICDH activity.

The effect of TQ on MDH activity was in accordance with several previous studies. A study conducted by Shao et al. [46] on the effects of arsenic, Cd, and lead on the growth and respiratory enzymes activities in wheat seedlings reported that the expression of MDH activity was enhanced at low concentrations of Cd. The improvement of MDH was found to help enhance Cd-chelating activity [47], which is in accordance with our results. Therefore, the increase of MDH activity in the roots after treatment with TQ may indicate a beneficial effect of this molecule in alleviating Cd toxicity in lentil seedlings by chelating activity.

Our results indicate that the use of TQ remarkably boosted the germination seeds’ growth, physiological parameters, regulated the antioxidant enzyme activities, and reduced the MDA and H_2_O_2_ contents in lentil seedlings (*Lens culinaris* L.) over the Cd treated plants. The increase in antioxidant enzyme activities and decrease in oxidative stress markers in lentil seedlings might be a stress tolerance mechanism of lentils under Cd treatment, and thymoquinone application helped the plants to boost this mechanism.

## 5. Conclusions

The results of our investigation showed that the toxicity of Cd affected all attributes of lentil seeds and seedlings, the overall growth parameters were decreased, and the enzymatic antioxidants modulated as a means of protecting the plants from further damage. However, the application of the TQ could mitigate cadmium-induced oxidative stress, germination seeds, and growth seedling inhibition, and enhance the cadmium tolerance in the seeds and seedlings lentils compared to Cd treated plants. Thus, thymoquinone may be considered as an effective as well as an alternative way to enhance the lentils seedlings’ tolerance against Cd.

However, further studies are needed to elucidate the mechanisms by which TQ helps the lentil plants to alleviate this element.

## Figures and Tables

**Table 1:** Effects of thymoquinone concentrations on lentil seeds and lentil seedlings traits affected by Cd stress.

	Control	Cd	10 µM Thy+ Cd	1 µM Thy+ Cd	0.1 µM Thy+ Cd
**GP, 24 h (%)**	68 ± 4.9 ^a,d^	47.20 ± 3.35 ^b^	60 ± 4.90 ^c^	70.40 ±2.19 ^d^	62.4 ± 2.2 ^a,c^
**GP, 48(%)**	86.40 ± 3.5 ^a^	71.20 ± 3.35 ^b^	89.60 ± 2.19 ^a,c^	88.80 ± 1.79 ^a,d^	92 ± 2.83 ^c,d^
**GP, 72 h (%)**	95.20 ± 3.3 ^a^	80 ± 6.32 ^b^	97.60 ± 3.58 ^a^	96 ± 4.90 ^a^	95.20 ± 3.35 ^a^
**GI (%)**	35.73 ± 1.5 ^c^	27.37 ± 0.78 ^b^	34.33 ± 1.16 ^a^	36.70 ± 1.02 ^c,d^	35.03 ± 0.6 ^a,d^
**SVI**	750.2 ± 64.0 ^a^	261.60 ± 38.7 ^b^	423.59 ± 36.75 ^c^	432.9 ± 42.2 ^c^	435.1 ± 41 ^c^
**Shoot length (cm)**	3.73 ± 0.39 ^c^	2.35 ± 0.36 ^b^	3.21 ± 0.29 ^c^	3.18 ± 0.34 ^c^	3.24 ± 0.31 ^c^
**Shoot fresh weight (mg)**	38.87 ± 4.8 ^a^	20.73 ± 4.35 ^b^	31.07 ± 4.35 ^c^	28.73 ± 3.33 ^c^	29.40 ± 3.14 ^c^
**Shoot dry weight (mg)**	4.01 ± 0.47 ^a^	2.04 ± 0.22 ^b^	3.67 ± 0.20 ^a^	3.26 ± 0.51 ^c^	3.15 ± 0.32 ^c^
**Roots length (cm)**	4.15 ± 0.60 ^a^	0.92 ± 0.14 ^b^	1.13 ± 0.10 ^b,c^	1.33 ± 0.13 ^c^	1.33 ± 0.10 ^c^
**Roots fresh weight (mg)**	45.87 ± 4.4 ^a^	9.87 ± 2.26 ^b^	15 ± 2.30 ^b^	18.47 ± 2.10 ^b^	16.27 ± 2.71 ^b^
**Roots dry weight (mg)**	3.91 ± 0.41 ^a^	1.15 ± 0.16 ^b^	1.87 ± 0.25 ^c^	2.01 ± 0.30 ^c^	1.73 ± 0.32 ^c^

GP: germinated seeds, GI: germination index and SVI: seedling vigor index. Control: treated only with water; Cd: treated with 300 µM Cd; 10 µM Thy+ Cd: treated with thymoquinone 10 µM + Cd (300 µM); 1 µM Thy+ Cd: treated with thymoquinone 1 µM + Cd (300 µM) and 0.1 µM Thy+ Cd: treated with thymoquinone 0.1 µM + Cd (300 µM). Each value represents the mean of four independent observations ±SD. Means with the same letter are not significantly different at the 5% probability.

## Data Availability

Not applicable.
